# py2DIC: A New Free and Open Source Software for Displacement and Strain Measurements in the Field of Experimental Mechanics †

**DOI:** 10.3390/s19183832

**Published:** 2019-09-05

**Authors:** Valeria Belloni, Roberta Ravanelli, Andrea Nascetti, Martina Di Rita, Domitilla Mattei, Mattia Crespi

**Affiliations:** 1 Geodesy and Geomatics Division, DICEA, Sapienza University of Rome, 00184 Rome, Italy; 2 Department of Structural and Geotechnical Engineering, Sapienza University of Rome, 00184 Rome, Italy; 3 Geoinformatics Division, Department of Urban Planning and Environment, KTH Royal Institute of Technology, 10044 Stockholm, Sweden

**Keywords:** Digital Image Correlation, free and open source software, full-field displacement and strain measurement

## Abstract

Thanks to the advances in computer power, memory storage and the availability of low-cost and high resolution digital cameras, Digital Image Correlation (DIC) is currently one of the most used optical and non-contact techniques for measuring material deformations. A free and open source 2D DIC software, named *py2DIC*, was developed at the Geodesy and Geomatics Division of the Sapienza University of Rome. Implemented in Python, the software is based on the template matching method and computes the 2D displacements and strains of samples subjected to mechanical loading. In this work, the potentialities of *py2DIC* were evaluated by processing two different sets of experimental data and comparing the results with other three well known DIC software packages *Ncorr*, *Vic-2D* and *DICe*. Moreover, an accuracy assessment was performed comparing the results with the values independently measured by a strain gauge fixed on one of the samples. The results demonstrate the possibility of successfully characterizing the deformation mechanism of the investigated materials, highlighting the pros and cons of each software package.

## 1. Introduction

The last few decades have been characterized by a continuous development of non-contact measurement techniques for displacement and deformation estimation [1,2,3,4,5,6,7]. Among these, Digital Image Correlation (DIC) is commonly applied to estimate full-field displacements and strains of structures or materials undergoing a mechanical loading by comparing temporal sequences of digital images acquired during the loading and deformation phenomena.

DIC can be performed in two (2D) or three (3D or stereo DIC) dimensions. The 2D DIC employs a single fixed camera and estimates displacements and deformations in a selected plane. Thus, 2D DIC is appropriate and useful only when the displacement and strain fields can be considered plane within the Area Of Interest (AOI). In this case, the image plane and the plane where deformations are estimated should be kept parallel and fixed during the acquisition of all the images, even if it has to be underlined that even significant misalignments up to 5 degrees with respect to this parallelism condition have a very small impact (within 0.5%) on the estimated displacements [4]. Alternatively, the 3D DIC is used when the whole 3D deformation field is looked for [1] but it needs two fixed cameras.

From an algorithmic point of view, subset-based DIC (local DIC) and finite element-based DIC (global DIC) are the two most commonly implemented approaches [8,9]. Local DIC estimates displacement and strain fields by correlating each subregion of the AOI at different stages of deformation to the corresponding subregion at the reference stage [10]. Thus, local DIC processes each node of the calculation grid independently, without imposing a displacement continuity to the global displacement fields [8]. On the other hand, global DIC usually discretizes the specified AOI with a finite element (FE) mesh and then tracks all these elements in the target image simultaneously. In this way, displacement continuity can be explicitly ensured between adjacent elements by the shared nodes of the FE mesh [8].

Nowadays, several DIC software solutions are available, both commercial and open source (see Section 2). However, the use of commercial software applications may be very expensive and they cannot be modified to fit specific requirements [11,12]. Alternatively, open source software can remarkably reduce costs and can be tailored to user needs [11,12]. For all these reasons, several research DIC codes were developed and made freely available to the scientific community. Among these research DIC software packages, a new, free and open source software (FOSS) for local 2D DIC, named *py2DIC*, was developed at the Geodesy and Geomatics Division of the Sapienza University of Rome (http://github.com/Geod-Geom/py2DIC/). Implemented in Python, the software can estimate 2D displacement and strain fields from an input set of images.

In this paper, by continuing the work started in References [11,13], *py2DIC* potentialities were evaluated. In particular, two different sets of experimental images were processed to estimate displacement and strain fields; the accuracy of the results was evaluated by comparing the estimated displacements with those computed from the strain values independently measured by a strain gauge fixed to one of the samples. Furthermore, the obtained displacements were also compared with those obtained through three renowned software applications: the well-known and mature open source software packages *Ncorr* and *DICe* and the commercial software *Vic-2D*, developed and sold by Correlated Solutions Inc, USA.

The paper is organized as follows: Section 2 gives a brief description of some of the available commercial and open source software applications; Section 3 presents the developed software *py2DIC* in detail; Section 4 illustrates the case studies and Section 5 discusses the obtained results and the corresponding comparisons. Finally, in Section 6 some conclusions are drawn and future prospects are outlined.

## 2. Commercial and Open Source DIC Software

Nowadays, different companies sell 2D and 3D DIC commercial software applications. Among these, Correlated Solutions, HOLO3, Dantec Dynamics, GOM, Image Systems, Imetrum, LaVision and Match ID are the most important ones [11]. At the same time, though, several research DIC codes are now made freely available to the scientific community: *Ncorr* (Georgia Institute of Technology, Atlanta, Georgia), *DICe* (Sandia National Laboratories, Albuquerque, New Mexico) and *YaDICs* (Laboratoire de Mécanique de Lille, Lille, France) are among the most popular ones [11], whereas several new Python applications, such as *py2DIC* (University of Rome La Sapienza, Rome, Italy), *pyxel* (Institut National des Sciences Appliquées de Toulouse, Toulouse, France), *pydic* (University of Limoges, Limoges, France) and *dolfin_dic* (École Polytechnique, Palaiseau, France) have been developed more recently. Among all these software solutions, the most mature and, at the same time, easy to install and used open source software packages *Ncorr* and *DICe* were selected, together with the commercial software *Vic-2D*, as term of comparison to evaluate *py2DIC* potentialities.

### 2.1. Commercial Software

Correlated Solutions, Inc. offers *Vic-2D* [14,15], a user friendly commercial software that uses optimized correlation algorithms to provide full-field displacements and strains for mechanical testing on planar specimens. Specifically, 2D displacements and strains are evaluated at every pixel subset within the AOI [16].

HOLO3 developed a licensed DIC software named CorreliSTC following the specifications defined by Airbus Group Innovations. The package can be used to measure 2D and 3D DIC displacements and strains and it is mainly applied in the aeronautics, automotive and energy industrial sectors [17].

Dantec Dynamics was founded during the 1950s and it is specialized in instrumentation for flow measurement and particle characterisation. Among the optical measurement systems it supports, it offers Q-400 to perform 3D DIC measurements [18].

GOM is an industrial manufacturer specialized in developing, producing and distributing optical measurement solutions and technologies for 3D coordinate measurement and deformation analysis. Among the developed technologies, ARAMIS software provides a non-contact and material-independent measuring system and solutions for full-field analysis [19].

Image Systems was born in 1999 and it offers a software named TEMA which allows for 2D and 3D measuring of full-field displacements and strains [20].

Imetrum is a company composed by a group of experts in non-contact measurements who operate in the field of rail bridge, road, geotechnical and structural monitoring. Among the products it sells, the Video Gauge^TM^ software offers the possibility to perform DIC to identify areas of high stress, crack opening or other discontinuities [21].

LaVision was founded in 1989 as a spin-off from Max Planck Institute and Laser Laboratory in Gottingen and it is focused on imaging systems, smart optical sensors, measurement technologies and software tools. It offers the 2D and 3D DIC system StrainMaster, a non-intrusive optical tool for shape and deformation analysis which combines the DIC algorithms with high quality hardware to offer an easy to use instrument for material analysis [22].

MatchID was founded by experts in the field of image correlation and it offers 2D and 3D DIC software [23].

### 2.2. Open Source Software

*Ncorr* [24] is an open source, freely available 2D subset-based Digital Image Correlation software. It was developed at the Georgia Institute of Technology and it is implemented in Matlab [24]. The software has a Graphical User Interface (GUI) and it provides plotting tools for figure creation [25]. It is used to estimate 2D displacement and strain fields starting from a set of speckle images [11].

*DICe* [26] is an open source DIC software developed by the Sandia National Laboratories, capable of computing full-field displacements and strains from a sequence of digital images. It is cross-platform and easy to use; package installers are available for Windows and Mac OS and instructions are provided to build the software on Linux. It has an intuitive GUI to perform 2D and 3D DIC. Furthermore, three analysis options can be selected: the subset-based full-field mode for local DIC, the global mode for finite element-based DIC and the tracking mode for trajectory tracking. The displacement and strain fields are not directly shown at the end of the analysis but the results can easily be post-processed in Paraview, a data analysis and visualization application [27].

*YaDICs* [28,29,30] was developed at the Laboratoire de Mécanique de Lille and runs on Linux operating systems. It is implemented in C++ and it is used for 2D and 3D solid and fluid kinematics field measurements. It allows the use of local and global methods by combining them at different pyramidal scales.

*pyxel* [31,32] is an open source global 2D DIC library for experimental mechanics applications. Developed at the Institut National des Sciences Appliquées de Toulouse, it is implemented in Python and is freely available for research and teaching.

*pydic* [33] is a free Python module for local 2D DIC based on OpenCV [34], one of the most used open source computer vision and machine learning library.

*dolfin_dic* [35] is a Python library for global 2D/3D DIC.

A synoptic illustration of the main features of the free and open source DIC software is given in Table 1.

## 3. py2DIC

**Figure 1:**
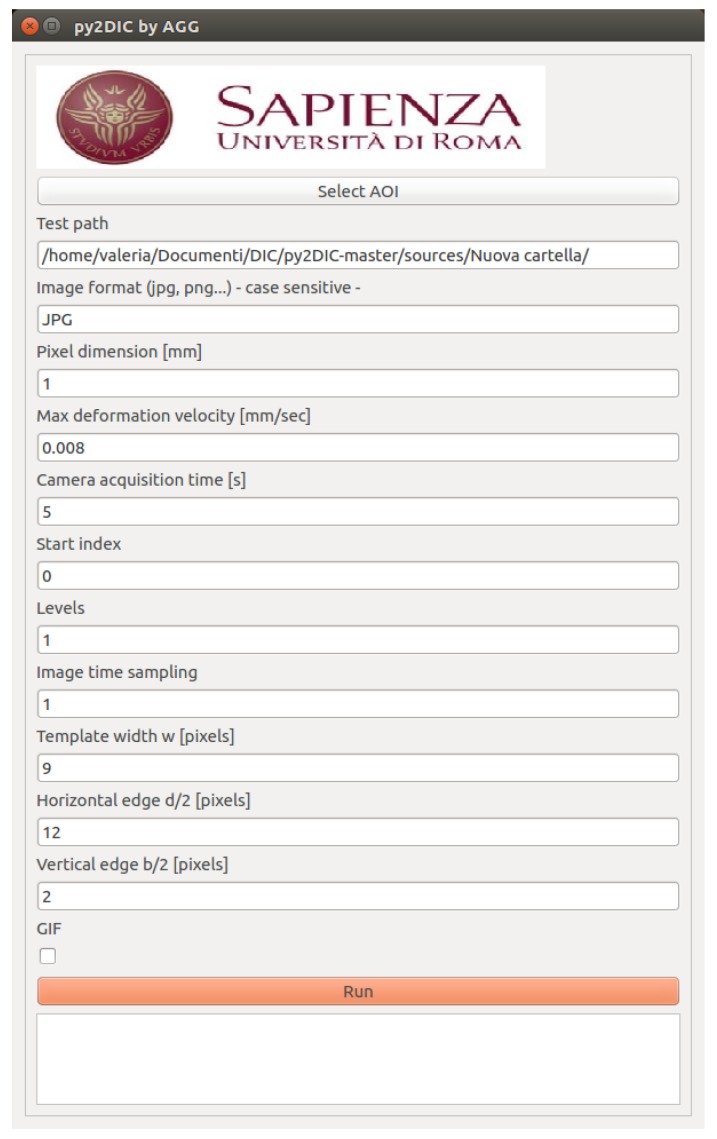
*py2DIC* GUI.

*py2DIC* is a free, open source and cross-platform local 2D DIC software based on the well-known template matching method. The software computes 2D displacements and strains of a sample by comparing one or more image pairs of its surface acquired in different steps of the deformation process. Implemented in Python, *py2DIC* has a GUI (Figure 1) and leverages the functionalities of OpenCV [34] computer vision library.

**Figure 2:**
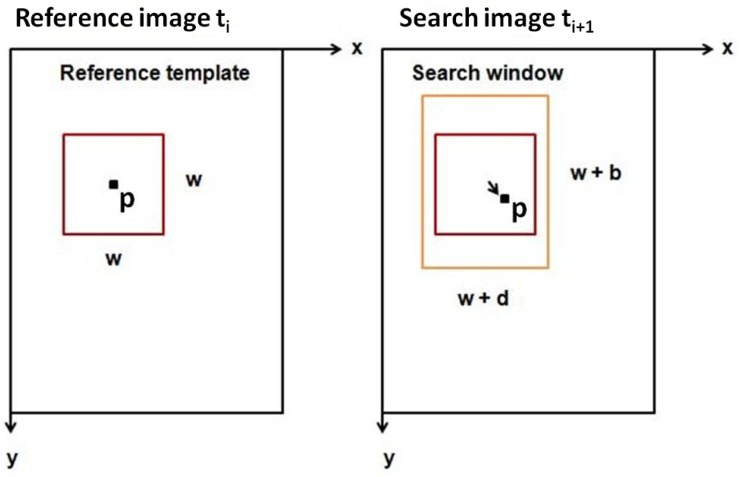
Scheme of the image pairs together with the reference template, the central pixel and the search window: the reference template width and height (*w*) and the dimensions of the edges (d/2,b/2) of the search window must be specified by the user in the GUI before starting the processing [13].

*py2DIC* template matching method involves different steps. At the beginning, a grid is defined to divide the AOI of the reference image into smaller areas. Then, the normalized cross-correlation index is computed through a convolution of a portion of the reference image (the reference template) with the corresponding larger subregion (the search window) in the search image (Figure 2); the sub-pixel resolution is reached by oversampling both the reference template and the research window using a bicubic interpolation. Finally, the maximum correlation coefficient value is used to detect the occurred displacement (u,v), being *u* and *v* respectively the components along the horizontal and vertical axes *x* and *y* [13].

Specifically, the procedure is implemented through the OpenCV function matchTemplate with the FNCC (Fast Normalized Cross-Correlation) similarity criterion (Equation (1)) [36]:

(1) $$\rho \left(x,y\right)=\frac{\begin{array}{c} \sum_{x^{\prime },y^{\prime }}\left(T^{\prime }\left(x^{\prime },y^{\prime }\right)\cdot I^{\prime }\left(x+x^{\prime },y+y^{\prime }\right)\right) \end{array}}{\sqrt{\begin{array}{c} \sum_{x^{\prime },y^{\prime }}T^{\prime }{\left(x^{\prime },y^{\prime }\right)}^{2} \end{array}\cdot \begin{array}{c} \sum_{x^{\prime },y^{\prime }}I^{\prime }{\left(x+x^{\prime },y+y^{\prime }\right)}^{2} \end{array}}}$$

where:

$$T^{\prime }\left(x^{\prime },y^{\prime }\right)=T\left(x^{\prime },y^{\prime }\right)-\frac{1}{wh}\sum_{x^{\prime \prime },y^{\prime \prime }}T\left(x^{\prime \prime },y^{\prime \prime }\right)$$

$$I^{\prime }\left(x+x^{\prime },y+y^{\prime }\right)=I\left(x+x^{\prime },y+y^{\prime }\right)-\frac{1}{wh}\sum_{x^{\prime \prime },y^{\prime \prime }}I\left(x+x^{\prime \prime },y+y^{\prime \prime }\right)$$

*T* denotes the reference template;
*I* denotes the search window;
ρ denotes the correlation coefficient;
*w* (width) and *h* (height) denote the reference template dimensions.

It is worth noticing that, in general, the reference template and the search window can be defined square or rectangular. In particular, *py2DIC* adopts a square reference template (w×w) and a rectangular search window (Figure 2) in order to consider the possible (also remarkable) differences between the displacements along the x and y directions. Once the analysis is performed along the first grid, independent staggered grids are considered to calculate displacements for every pixel of the AOI, increasing in this way the resolution of the computed displacement fields.

Regarding the strains, a cubic spline-based filter or a Gaussian filter can be applied to smooth the displacements estimated at each pixel of the considered staggered grids and to reduce their noise. Both the filters, implemented starting from the SciPy library functions, are able to handle missing data or masked area. Then, the obtained smoothed displacements are differentiated using the centered difference approximation, for which the weights (up to the eighth order of accuracy) are generated following the approach described in Reference [37]. Finally, the Green Lagrangian strains are computed according to the following equations:

(2a) $$\begin{array}{c} E_{xx}=\frac{1}{2}(2\frac{\partial u}{\partial x}+{\left(\frac{\partial u}{\partial x}\right)}^{2}+(\frac{\partial v}{\partial x}){}^{2}) \end{array}$$

(2b) $$\begin{array}{c} E_{xy}=\frac{1}{2}\left(\frac{\partial u}{\partial y}+\frac{\partial v}{\partial x}+\frac{\partial u}{\partial x}\frac{\partial u}{\partial y}+\frac{\partial v}{\partial x}\frac{\partial v}{\partial y}\right) \end{array}$$

(2c) $$\begin{array}{c} E_{yy}=\frac{1}{2}\left(2\frac{\partial v}{\partial y}+{\left(\frac{\partial u}{\partial y}\right)}^{2}+{\left(\frac{\partial v}{\partial y}\right)}^{2}\right) \end{array}$$

where:

$E_{xx}$, $E_{xy}$ and $E_{yy}$ are the Green Lagrangian strains;
$\frac{\partial u}{\partial x}$, $\frac{\partial u}{\partial y}$, $\frac{\partial v}{\partial x}$, $\frac{\partial v}{\partial y}$ are the displacement gradients.

The analysis is repeated for different pairs of images captured at different temporal steps: the results are the accumulated displacement and strain fields for each pair of the processed images.

## 4. Case Studies

To investigate the potentialities of *py2DIC*, two different examples were processed.

### 4.1. Plate Hole DIC Challenge

**Figure 3:**
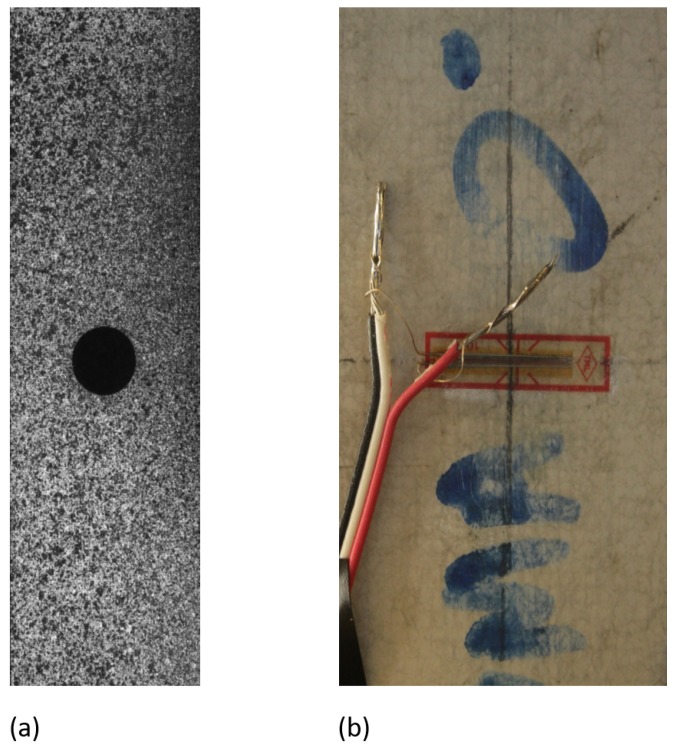
(**a**) “plate hole” sample image (**b**) GFRP sample image.

The first set of input images was selected from the Society for Experimental Mechanics (SEM) 2D-DIC challenge simulated datasets [38,39] (Bethel, CT, USA). The DIC challenge provides common image datasets that can be used to validate and improve both commercial and academic DIC software solutions. Specifically, in order to make a comparison between *py2DIC*, *Ncorr*, *Vic-2D* and *DICe*, the “plate hole” sample images (Sample 12) from SEM DIC challenge were analysed. Sample 12 is an experimental set of 12 images (resolution of 400 × 1040 pixels) of a steel plate with a hole in the middle being loaded in tension (Figure 3). Regarding the specimen, a painted speckle pattern was used during the tensile test. As this is an experimental image series, there are no ground truth data; this is the reason why it is useful for round-robin type tests where different codes for DIC implementation are compared [39].

### 4.2. Tensile Test of Glass Fiber Reinforced Polymer Samples

The second set of input images was acquired during an experimental campaign performed in the Laboratory of Structural Engineering of the Department of Structural and Geotechnical Engineering at the Sapienza University of Rome. The tests aimed at the study of the mechanical properties of standard Glass Fiber Reinforced Polymer (GFRP) specimens obtained from GFRP beams. The specimen dimensions are reported in Table 2.

During the campaign the specimen was subjected to tensile test by means of a servo-hydraulic universal testing machine (ZwickRoell, Berlin, Germany). The test involved placing the GFRP specimen in the grips of the testing machine and slowly extending it until failure. Specifically, the upper part of the specimen was fixed to two steel crossheads and the pull load was applied at the bottom part of the specimen using a displacement-control protocol system (0.5 mm/min). During the process the elongation of the gauge section was recorded against the applied force. A metric reference was used to calculate the pixel dimension necessary to perform the conversion pixels-to-unit length for the measured displacement fields. In order to measure the specimen local deformations, vertical and horizontal standard strain gauges were fixed on the sample surface, where no speckle pattern was applied (Figure 3). Indeed, it is worth noticing that one of the aim of the software comparison is also to test the responses of the different matching criteria implemented in the compared applications which, in general, should be able to deal with in-situ measures performed outside the elementary lab conditions, without the possibility of adding artificial speckle.

**Figure 4:**
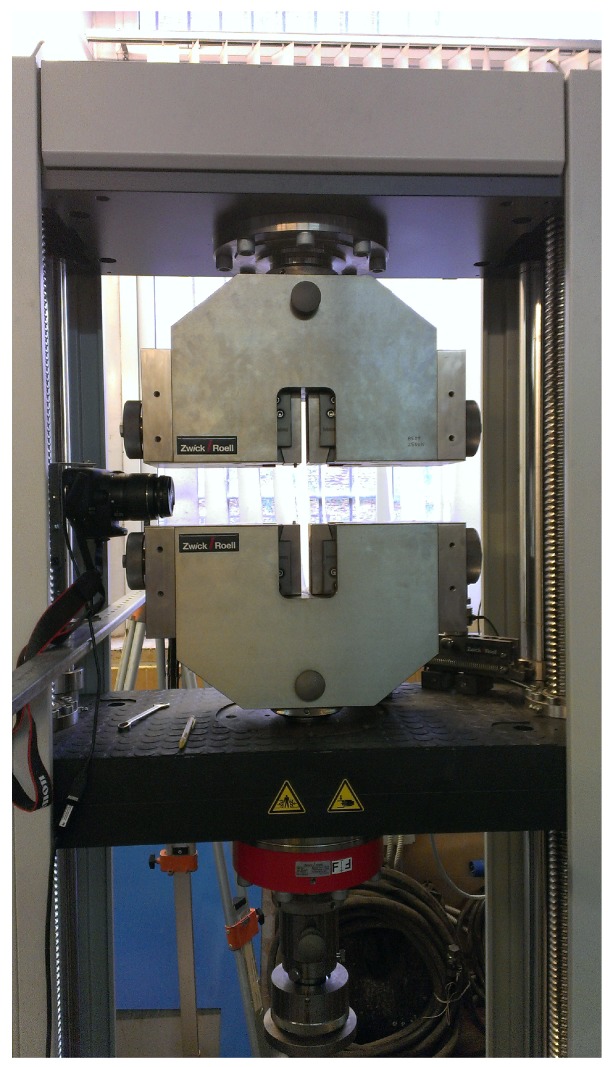
Experimental setup.

Furthermore, a Canon EOS 1200D camera (Canon, Bangkok, Thailand) was placed around 20 centimetres far from the sample on a metallic bar fixed over two tripods to avoid vibrations during the loading tests. The camera was connected to a standard laptop and the EOS Digital Solution Disk Software 31.4A (Canon, Bangkok, Thailand) was used to acquire images with a time sampling of one acquisition every 5 s and a resolution of 3456×5184 pixels. The experimental setup used during the campaign is shown in Figure 4.

## 5. Results and Discussion

*py2DIC* was validated using the two mentioned sets of images which were processed to obtain horizontal (*u*) and vertical (*v*) displacement fields. For the first image set, also the Green Lagrangian strain fields ($E_{xx}$, $E_{xy}$ and $E_{yy}$) were computed. Furthermore, the same sets of images were used as input for both *Ncorr*, *Vic-2D* and *DICe* software applications. The differences between the raw displacements (not smoothed) computed with *py2DIC* and those obtained with each of the compared software were also calculated as follows:

(3) $$\Delta u=u_{i,j}^{py2DIC}-u_{i,j}^{Vic-2D/Ncorr/DICe}\;\Delta v=v_{i,j}^{py2DIC}-v_{i,j}^{Vic-2D/Ncorr/DICe}$$

where *i* and *j* are the row and column pixel locations, respectively.

Referring to displacement differences Δu and Δv (Equation (3)), a global approach was performed for all the points of the grid, considering the following standard statistical parameters in order to summarize the error of full-field displacements:

- $\bar{\Delta u}$$\bar{\Delta v}$: mean value of the horizontal and vertical displacement differences
- $\tilde{\Delta u}$$\tilde{\Delta v}$: median value of the horizontal and vertical displacement differences
- Std.Dev: standard deviation of the horizontal and vertical displacement differences where N is the number of data points:
  $\sigma_{u}=\sqrt{\frac{1}{N}\sum_{i,j}{\left(\Delta u-\bar{\Delta u}\right)}^{2}}$$\sigma_{v}=\sqrt{\frac{1}{N}\sum_{i,j}{\left(\Delta v-\bar{\Delta v}\right)}^{2}}$
- RMSE: Root Mean Square Error of the horizontal and vertical displacement differences
  $RMSE_{u}=\sqrt{{\left(\sigma_{u}\right)}^{2}+{\left(\bar{\Delta u}\right)}^{2}}$$RMSE_{v}=\sqrt{{\left(\sigma_{v}\right)}^{2}+{\left(\bar{\Delta v}\right)}^{2}}$
- NMAD: Normalized Median Absolute Deviation
  $NMAD_{u}=1.4826*median|\Delta u-\tilde{\Delta u}|$$NMAD_{v}=1.4826*median|\Delta v-\tilde{\Delta v}|$
- LE68: Linear error with 68% of probability
- LE95: Linear error with 95% of probability

Finally, in order to examine the results more closely, the *u* and *v* displacement values obtained from *py2DIC*, *Ncorr*, *Vic-2D* and *DICe* were plotted along vertical sections.

### 5.1. Plate Hole DIC Challenge Displacement Field Comparison

The first and the last image of the experimental set with a resolution of 400×1040 pixels were processed using a local approach for all the software applications. For this set of experimental images, the horizontal and vertical displacement values were calculated in pixels, as no conversion pixels-to-unit length was available. *py2DIC* results were calculated at every pixel of the considered images, using a 11×11 pixel template, a step size of 1 pixel and oversampling factor of 20 and 10 for the *u* and *v* displacement computation respectively.

**Figure 5:**
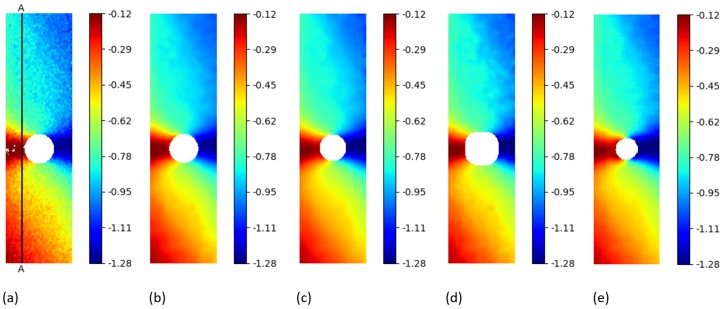
u displacements (px) for the “plate hole” sample from SEM DIC challenge obtained from DIC technique. (**a**) *py2DIC* raw results (**b**) *py2DIC* smoothed results using Gaussian filter (**c**) *Ncorr* results (**d**) *Vic-2D* results (**e**) *DICe* results.

**Figure 6:**
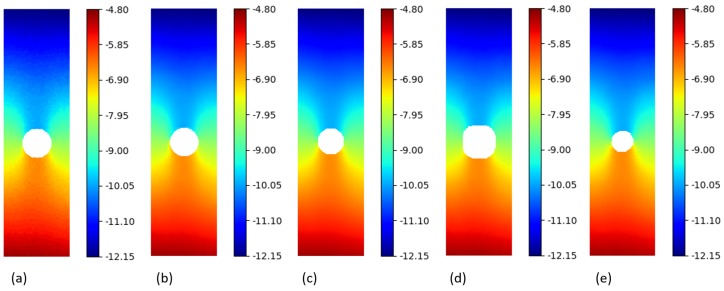
v displacements (px) for the “plate hole” sample from SEM DIC challenge obtained from DIC technique. (**a**) *py2DIC* raw results (**b**) *py2DIC* smoothed results using Gaussian filter (**c**) *Ncorr* results (**d**) *Vic-2D* results (**e**) *DICe* results.

Firstly, the *py2DIC* and *Ncorr* comparison was performed; the obtained displacement fields are shown in Figure 5 and Figure 6. *Ncorr* returned the displacements on a 2-pixel spaced grid.

Regarding this comparison, the results are in reasonable agreement with each other. In fact, starting from the displacement differences of the two software, the above mentioned statistical parameters were computed and they are reported in Table 3. The quite low mean differences highlight the absence of significant systematic differences in both the directions and the equality of mean and median differences witnesses the absence of outliers. The substantial equality of Std. Dev’s., NMAD’s and LE68’s confirms the normal distribution of the differences. Furthermore, the RMSE amounts to few hundredths of a pixel. At the same time, anyway, it can be noticed that the statistics of the vertical displacements are slightly worse than the horizontal ones.

Secondly, the differences between the displacements computed using *py2DIC* and *Vic-2D* were analyzed. *Vic-2D* returned the displacements for every pixel of the considered images, as for *py2DIC*.

The obtained displacement fields are shown in Figure 5 and Figure 6, whereas Table 4 reports the statistics of the differences among the displacements computed with the two software applications. Quite similar results to the previous comparison were obtained in this case too, together with the slightly worse behaviour of *py2DIC* on the vertical displacements. Furthermore, it is highlighted in Figure 6 that *Vic-2D* is not able to compute the displacements near the hole and this is probably due to the handling of the Nan values.

Thirdly, *py2DIC* results were compared with those obtained using *DICe* in terms of displacement fields (Figure 5 and Figure 6) and statistical analysis ( Table 5). *DICe* returned the displacements at every pixel of the considered images, as for *py2DIC*.

Again, the quite low mean differences highlight the absence of significant systematic errors in both the directions; the normal distribution of the differences and the slightly worse behaviour of vertical displacements are also confirmed in this test.

**Figure 7:**
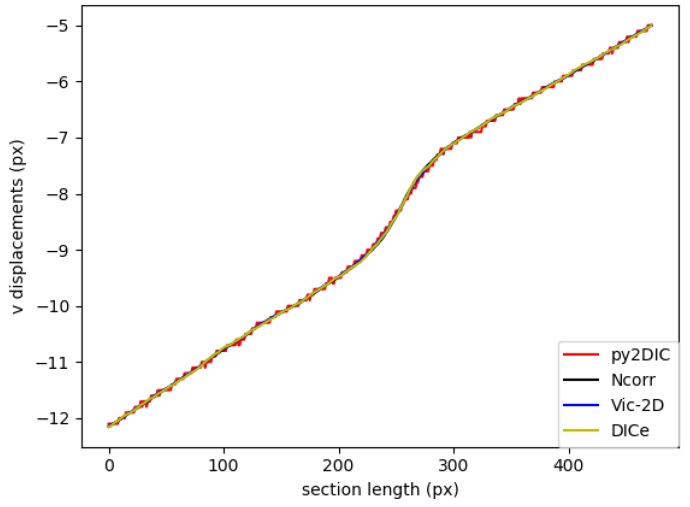
Comparison of v displacement along the section AA among *py2DIC*, *Ncorr*, *Vic-2D* and *DICe*.

Finally, in order to locally characterize the potentialities of *py2DIC*, a vertical section AA ( Figure 5) was cut on the displacement data obtained with the developed software and the three reference applications. The results are shown in Figure 7 and highlight how *py2DIC* raw displacements well follow the trends of the compared software applications, at the level of few hundredths of a pixel, even if, of course, they are less smooth.

### 5.2. Plate Hole DIC Challenge Strain Field Comparison

**Figure 8:**
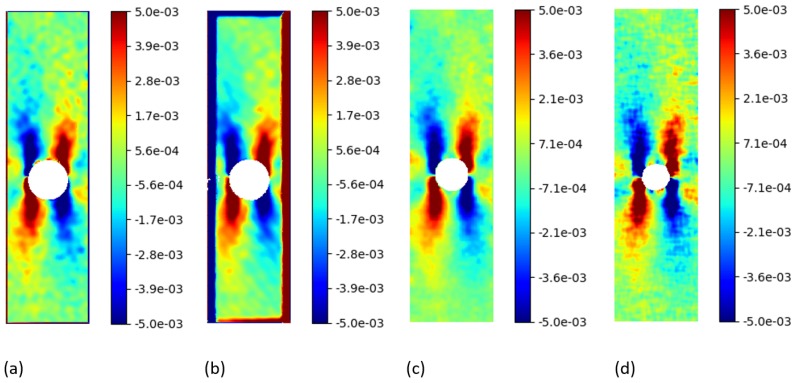
$E_{xy}$ Green Lagrangian strains for the “plate hole” sample from SEM DIC challenge obtained from DIC technique. (**a**) *py2DIC* results using Gaussian filter (**b**) *py2DIC* results using spline filter (**c**) *Ncorr* results (**d**) *DICe* results.

**Figure 9:**
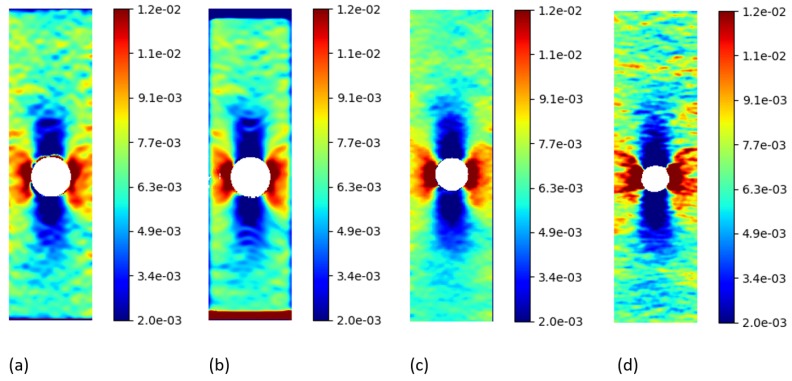
$E_{yy}$ Green Lagrangian strains for the “plate hole” sample from SEM DIC challenge obtained from DIC technique. (**a**) *py2DIC* results using Gaussian filter (**b**) *py2DIC* results using spline filter (**c**) *Ncorr* results (**d**) *DICe* results.

Starting from the obtained displacement fields, the Green Lagrangian strains $E_{xx}$, $E_{xy}$, $E_{yy}$ were computed using the open source software *py2DIC*, *Ncorr* and *DICe*. Regarding *py2DIC*, in order to reduce the noise, both Gaussian and spline smoothing methods were applied and then the convolution procedure was performed. As for *Ncorr*, a strain radius equal to 5 pixels was chosen to define a local group of displacement data points and perform a least squares plane fit, as proposed in Reference [40]. This method is used to estimate the plane parameters and retrieve the displacement gradients and the subsequent Green Lagrangian strains. Finally, a gauge size of 15 pixels was used to define a displacement subset for *DICe* strain computation. The obtained $E_{xy}$ and $E_{yy}$ strain fields are reported in Figure 8 and Figure 9. The visual comparisons among *py2DIC* and the other software packages are globally consistent, being evident a good agreement with *Dice* and *Ncorr*. Note that the numerical comparison is not at all straightforward, since each software computes the strain field over a proper grid; so, it would have been necessary an additional interpolation, which would have impacted the comparison.

### 5.3. Tensile Test of GFRP Sample Displacement Field Comparison

For the tensile test image pair, a local approach was used for *py2DIC*, *Ncorr* and *Vic-2D* processing, whereas a global approach was tested using *DICe*. In this case the results were calculated in millimetres thanks to the availability of the pixels-to-unit length conversion factor (as approximation, the pixel dimension was considered squared and constant throughout the image).

**Figure 10:**
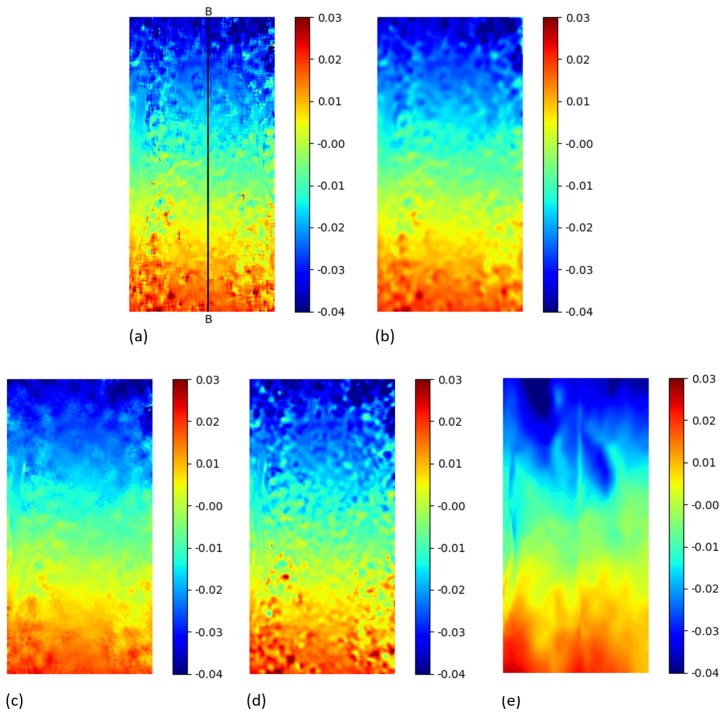
u displacements (mm) for the tensile test of GFRP sample obtained from DIC technique. (**a**) *py2DIC* raw results (**b**) *py2DIC* smoothed results using Gaussian filter (**c**) *Ncorr* results (**d**) *Vic-2D* results (**e**) *DICe* results [11].

**Figure 11:**
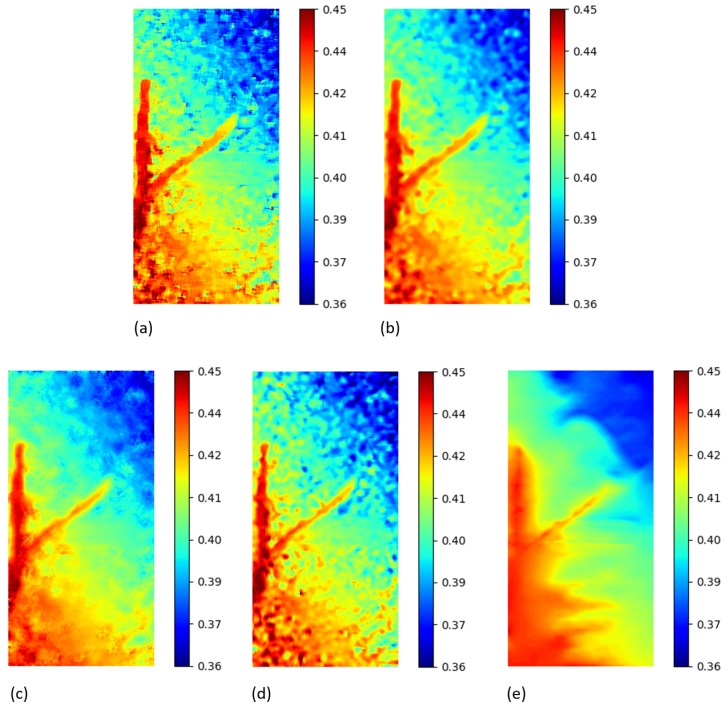
v displacements (mm) for the tensile test of GFRP sample obtained from DIC technique. (**a**) *py2DIC* raw results (**b**) *py2DIC* smoothed results using Gaussian filter (**c**) *Ncorr* results (**d**) *Vic-2D* results (**e**) *DICe* results [11].

**Figure 12:**
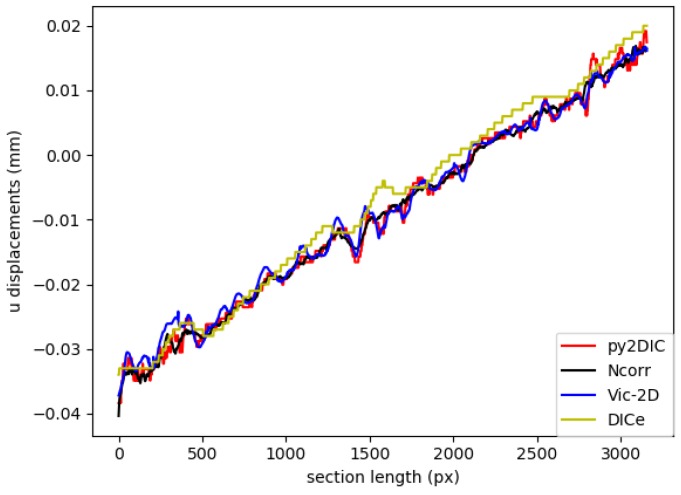
Comparison of u displacement along the section BB among *py2DIC*, *Ncorr*, *Vic-2D* and *DICe*.

**Figure 13:**
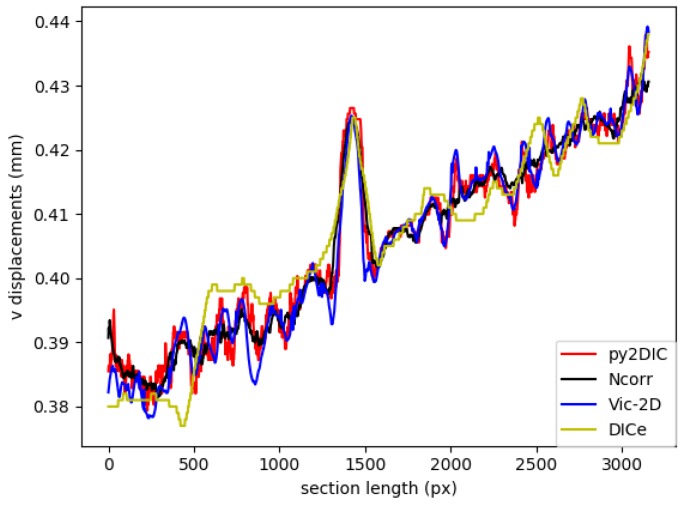
Comparison of v displacement along the section BB among *py2DIC*, *Ncorr*, *Vic-2D* and *DICe*.

Specifically, *py2DIC* horizontal and vertical displacements were computed using a 65×65 pixel template, a step size of 1 pixel and oversampling factor of 20 for both the displacement directions. Then, the displacement differences and the correspondent statistical parameters were calculated for the comparisons with *Ncorr*, *Vic-2D* and *DICe*. In particular, the results from the *DICe* FE approach were interpolated on a regular grid to facilitate the comparison with *py2DIC*. The obtained displacement maps are reported in Figure 10 and Figure 11. Furthermore, also for this set of images a local comparison was performed on the vertical section BB shown in Figure 10. The results are shown in (Figure 12 and Figure 13). It is worth noticing that, as for the former set of images, *py2DIC*, *Vic-2D* and *DICe* returned the displacements for every pixel, while *Ncorr* computed the displacements on a 2-pixel spaced grid. Table 6, Table 7 and Table 8 refer to the comparison respectively with *Ncorr*, *Vic-2D* and *DICe* in terms of statistical parameters.

Also for this set of images, it is possible to observe a very good correspondence between the *u* and *v* displacements computed by *py2DIC* and those generated using *Ncorr*, *Vic-2D* and *DICe*. Neither biases, no outliers were highlighted and again a normal distribution of the differences was found. Also, the analysis of the values of the statistical parameters highlights few microns agreement with respect to all the compared software applications. Moreover, also in this case, the comparison is slightly worse for what regards the vertical displacements. Finally, the vertical section comparisons show how software displacements are in good agreement with each other even if, in this case, the adoption of *DICe* global approach leads to slightly different results.

### 5.4. Tensile Test of GFRP Sample Strain Gauge Comparison

**Figure 14:**
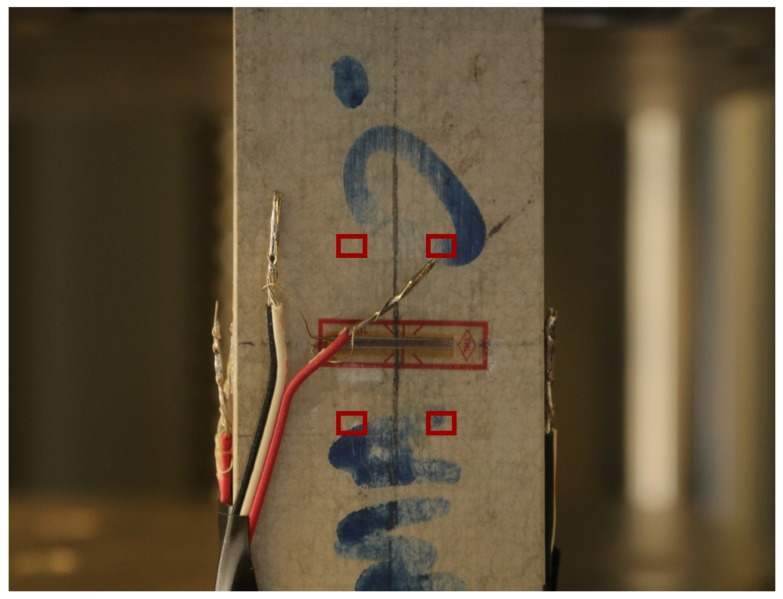
Strain gauge setup.

For the tensile test of the GFRP sample, *py2DIC* results were also compared to strain gauge measurements. Specifically, a strain gauge was placed horizontally on the sample side facing the camera, as shown in Figure 14 and *py2DIC* displacements were calculated and spatially averaged on the points corresponding to the left and right extremities of the device.

**Figure 15:**
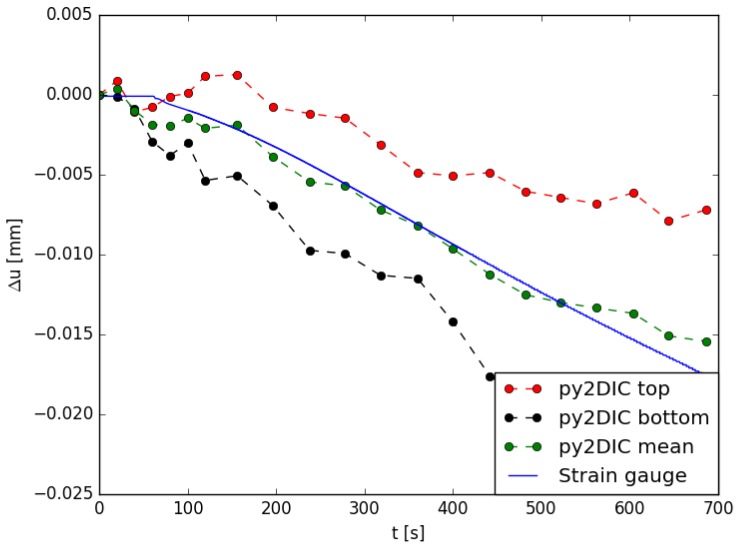
Comparison among *py2DIC* horizontal displacements and strain gauge measurements [13].

Then, since the sample central section was covered by the wires of the strain gauge, the difference between the displacements observed in these two positions was calculated at the bottom and top part of the strain gauge, respectively (Figure 14). Finally, these two displacement differences were averaged at different time steps and compared with the displacements inferred using the strain gauge. The results are shown in Figure 15 and highlight how the averaged *py2DIC* displacements well follow the strain gauge reference trend: the RMSE of *py2DIC* displacements computed with respect to the strain gauge measurements is of the order of some microns [13].

## 6. Conclusions and Prospects

*py2DIC*, a free and open source 2D DIC software was developed at the Geodesy and Geomatics Division of the Sapienza University of Rome. The potentialities of the developed software were investigated by processing two different sets of experimental images and comparing the results with those supplied by the three well known software packages, one commercial (*Vic-2D*) and two free and open source (*Ncorr* and *DICe*) and with those coming from independent measurements by a strain gauge fixed on one of the samples. The first set of images was selected from the SEM DIC challenge simulated datasets; the second one was acquired during an experimental campaign performed in collaboration with the Lab of Structural Engineering of the Sapienza University of Rome and it is available at http://github.com/Geod-Geom/py2DIC/tree/master/LabTest together with the strain gauge measurements. The obtained results point out the very good *py2DIC* performances in successfully characterizing the deformation mechanism of material subjected to mechanical loading: the new software is indeed able to estimate displacements with an agreement at the level of few hundredths of a pixel or few microns; also, the compared strain fields are globally consistent, even if in this case only a graphical comparison was possible, since each software adopts a proper grid. In conclusion, *py2DIC* represents a reliable free and open source alternative software for 2D DIC applications in the field of solid mechanics.

In the future, this research project will aim to investigate the potentialities of *py2DIC* outside the elementary lab conditions, in order to bring the methodology and the software at a full 2D operational level in the widest practical cases, releasing as much as possible the acquisition constraints related to the testing setup. Furthermore, it would be worth implementing the 3D DIC method, to also characterize the 3D deformations which can occur during the loading tests. Finally, in order to increase the computational efficiency of the software, a pyramidal oversampling approach will be implemented, along with a multi-threaded computation of the displacements over all the staggered grids.

References

## Figures and Tables

**Table 1:** Open-source Digital Image Correlation (DIC) software features.

Software	2D/3D	Approach	Language	OS	Code Repository
DICe	2D/3D	Local/Global	C++	Cross-platform	https://github.com/dicengine/dice
dolphin_dic	2D/3D	Global	Python	Cross-platform	https://bitbucket.org/mgenet/dolfin_dic/src/master/
Ncorr	2D	Local	Matlab	Linux/Windows	https://github.com/justinblaber/ncorr_2D_matlab
pydic	2D	Local	Python	Cross-platform	https://gitlab.com/damien.andre/pydic
pyxel	2D	Global	Python	Cross-platform	https://github.com/jcpassieux/pyxel
**py2DIC**	**2D**	**Local**	**Python**	**Cross-platform**	**http://github.com/Geod-Geom/py2DIC/**
YaDICs	2D/3D	Local/Global	C++	Linux	http://yadics.univ-lille1.fr/wordpress/

**Table 2:** Sample dimensions.

Width (mm)	Height (mm)	Thickness (mm)
30	120	8

**Table 3:** Comparison with *Ncorr*: statistical parameters of displacement differences.

(px)	Δu py2DIC-Ncorr	Δv py2DIC-Ncorr
Mean	−0.0023	−0.0027
Median	−0.0023	−0.0027
Std.Dev	0.0302	0.0389
RMSE	0.0303	0.0390
NMAD	0.0298	0.0416
LE68	0.0301	0.0400
LE95	0.0586	0.0740

**Table 4:** Comparison with *Vic-2D*: statistical parameters of displacement differences.

(px)	Δu py2DIC-Vic-2D	Δv py2DIC-Vic-2D
Mean	−0.0048	−0.0050
Median	−0.0050	−0.0049
Std.Dev	0.0303	0.0381
RMSE	0.0307	0.0385
NMAD	0.0298	0.0410
LE68	0.0302	0.0396
LE95	0.0588	0.0721

**Table 5:** Comparison with *DICe*: statistical parameters of displacement differences.

(px)	Δu py2DIC-DICe	Δv py2DIC-DICe
Mean	−0.0054	−0.0033
Median	−0.0063	−0.0033
Std.Dev	0.0339	0.0411
RMSE	0.0344	0.0412
NMAD	0.0338	0.0431
LE68	0.0340	0.0422
LE95	0.0657	0.0785

**Table 6:** Comparison with *Ncorr*: statistical parameters of displacement differences.

(mm)	Δu py2DIC-Ncorr	Δv py2DIC-Ncorr
Mean	−0.0001	0.0002
Median	−0.0001	0.0002
Std.Dev	0.0035	0.0055
RMSE	0.0035	0.0055
NMAD	0.0023	0.0041
LE68	0.0026	0.0044
LE95	0.0074	0.0112

**Table 7:** Comparison with *Vic-2D*: statistical parameters of displacement differences.

(mm)	Δu py2DIC-Vic-2D	Δv py2DIC-Vic-2D
Mean	−0.0001	0.0004
Median	−0.0000	0.0002
Std.Dev	0.0034	0.0058
RMSE	0.0034	0.0058
NMAD	0.0022	0.0039
LE68	0.0025	0.0043
LE95	0.0069	0.0114

**Table 8:** Comparison with *DICe*: statistical parameters of displacement differences.

(mm)	Δu py2DIC-DICe	Δv py2DIC-DICe
Mean	−0.0003	−0.0004
Median	−0.0005	−0.0002
Std.Dev	0.0051	0.0075
RMSE	0.0051	0.0075
NMAD	0.0043	0.0067
LE68	0.0044	0.0068
LE95	0.0106	0.0150
